# Menopause and the role of physical activity – The views and knowledge of women aged 40–65

**DOI:** 10.1177/20533691241235273

**Published:** 2024-02-23

**Authors:** David Wasley, Samantha Gailey

**Affiliations:** Cardiff School of Sport and Health Sciences, 11352 Cardiff Metropolitan University, Cardiff, UK

**Keywords:** Menopause, perimenopause, post-menopause, awareness, education, physical activity, exercise

## Abstract

Menopause marks the end of female reproductive capacity. It is defined as the point after cessation of the menstrual cycle for 12 months (Nursat et al., 2008). Awareness about menopause has increased over the last decade, yet studies have shown that women still lack knowledge regarding the subject. Likewise, awareness of women between the age of 40–65 on the potential role of physical activity prior to and during menopause in women is unclear. Women (*n* = 162) aged 40–65 years completed a survey rating their knowledge, answered fact-based questions and reported their experiences of menopause. Their levels of, and beliefs on, the role physical activity on symptoms and menopause associated disease risk were also collected. Women reported their confidence in their current knowledge level at 67% reflecting 37% higher rating than an estimate of their knowledge 10 years ago. Their factual knowledge score was 56%. Knowledge was primarily gained through friends and family and almost half (46%) had not spoken to a healthcare professional. Frustration was expressed with lack of knowledge and support of healthcare and others. Women using HRT (44%) had mixed attitudes towards its role. A high proportion were active and felt that physical activity can help manage symptoms and impact long-term health consequences of menopause. Menopause education strategies for women, healthcare professionals and others need to be improved. Lack of education may be causing women to struggle and feel negatively towards this life stage. Physical activity was viewed positively for the symptoms and a treatment during menopause and long-term health.

## Introduction

Natural menopause occurs in women at an average age of 51 years and is diagnosed after cessation of the menstrual cycle for 12 months without any other physiological or biological explanation, with that transition time being termed perimenopause.^1^ It involves a loss of ovarian function due to reducing estrogen levels. This lack of estrogen compromises physical and mental well-being, resulting in many bothersome symptoms such as hot flushes, night sweats, weight gain, anxiety, loss of libido and results in a loss of fertility.^2^

Defining the stages of menopausal transition has been problematic due to the lack of consistent definition which has complicated the synthesis and interpretations of research findings.^3^ Early definitions from the World Health Organisation^4^ and from the International Menopause Society^5^ were deemed unsatisfactory regarding dealing with the menopausal transition.^6^ When the Stages of Reproductive Aging Workshop (STRAW) staging system was released (2011), it enabled a consistent classification of menopause status to be used as a clinical tool for women and their healthcare providers.^6^ Importantly, menopause and being post-menopausal is associated with an increased risk of chronic diseases such as osteoporosis, cardiovascular disease and type 2 diabetes and cardiovascular risks increase to those of men.^7^ Additionally, historical education regarding menopause has been lacking which has resulted in generations of women having limited knowledge on symptoms and treatment and feeling confused and unsure on how to deal with the menopausal transition.^2,8,9^

Over the last decade in the United Kingdom, there have been improvements in providing menopause education for women and healthcare professionals.^10^ In part, this may be built on the introduction in 2015 of the first National Institute for Health and Care Excellence (NICE)^11^ guidelines concerning menopause. Munn et al.^12^ suggested the publication encouraged the acceptance and importance of menopause within healthcare. However, Harper et al.^9^ reported that that 60% of 947 UK respondents felt uninformed and had a lack information regarding menopause.

While many women still report a lack of information on menopause, they may also lack information on lifestyle behaviours that could influence of going through menopause. Studies have shown that the risk of developing chronic post-menopausal ill health, for example, osteoporosis/diabetes can be lowered through regular physical activity^13^ (PA). Likewise, exercise may attenuate some of the experiences of moving through perimenopause to menopause.^14^ Thus, this study assessed the knowledge, sources of knowledge and awareness of menopause amongst women between the ages of 40 and 65 who are pre-, peri- and post-menopausal and gain insight into their beliefs about the role of exercise.

## Methods

### Participants

Participants were 162 women between the age of 40 and 65 (mean age = 52, SD = 5.1) reporting no serious illnesses nor had gone through medically induced menopause provided complete surveys. A further 56 respondents gave consent but did not respond to the first question ‘rate your current knowledge of menopause’ and a further 13 had incomplete randomly distributed data and removed from the study. Education level attained (or equivalent) were 10% O-level/CSE, 16% A level, 35% degree, 36% masters and 3% PhDs, while the main occupations represented were medical (33%), management (35%) and education (15%) with others ranging from student (*n* = 1) to retired (*n* = 5). The study was approved by the University Ethics Panel.

### Materials

This study used an online questionnaire, designed on the Qualtrics XM platform with four parts. The first part obtained descriptive details of the participant including age, occupation and highest educational level achieved. The second part assessed knowledge on menopause with two general questions: ‘How would you describe your current knowledge level on menopause’ and ‘How would you describe your knowledge level on menopause 10 years ago’. Responses were obtained using visual analogy sliders from 0 ‘very poor’ to 100 ‘very good’. Subsequent questions addressed: where participants sourced, if any, menopause information; knowledge based open and multiple-choice items on symptoms and health implications selected from two menopause knowledge surveys, one of which is validated and the second based on the work of the University of Rochester NY.^10,15^ Other questions assessed awareness and participants willingness to engage with others including health professionals on the topic of menopause and their overall view on the period of menopause in their life. Aspects of knowledge were individually explored and summed to calculate an overall score. The third part allowed participants to respond to questions according to what category of menopause they believed they were at pre-, peri- or post-menopause. A description of each was provided at this point but participants were unable to return to previous sections of the survey. The final section focussed on PA and exercise. It asked about their rating of PA level, exercise reasons, barriers, and experiences if any. Knowledge and beliefs on how exercise may impact menopause based on the work of Mishra et al.^13^ and Daley and Thomas^16^ were then addressed. Participants were provided with links to educational sources and thanked for their completing the survey.

### Procedure

The survey content and its administration were piloted on three levels with revisions adopted where required: the authors completed the survey for functionality, logic and question; three post-menopausal women completed the online survey draft and were interviewed after completion; revisions were made, and the final survey was piloted with two women.

Recruitment to the study was achieved through various approaches: snowball, social media, WhatsApp groups and the University Participant Panel. Invite to study contained brief information on the study, who was eligible and access to the survey via a Qualtrics XM link. Detailed information and consent forms were embedded at the beginning to ensure eligibility and informed consent criteria were met. Participants were free to withdraw at any point and post survey withdrawal forms prior to data analysis were made available. The survey was live between January 2023 and March 2023.

### Data analysis

The quantitative data collected was coded, double entered, cleaned and analysed using SPSS version 29 (IBM Inc). Tests for normality showed the data to be non-parametric for all continuous variables (Kolonoff-Smirnov *n* > 50 *p* < .05). Averages are reported for continuous variables and frequency counts for categorical variables. Scores for current knowledge rating, knowledge and previous knowledge level were calculated for the sample and compared using Freidman and Mann–Whitney tests (*p*-value of less than 0.05). Based on self-identification, the sample was split into 3 categories: Pre-, peri- and post-menopausal and the differences between groups were compared using Kruskal–Wallis tests. Qualitative data were analysed via thematic analysis and Braun and Clarke’s^17^ 6-stage method. Stages involved; familiarisation with the data, generating initial codes, searching for themes, reviewing themes, defining and naming themes and then producing the report.^17^

## Results

### Menopause knowledge

There was a significant difference in the overall model (Friedman χ^2^_(2)_ = 197.255, *p* < .001) between women’s rating of their current knowledge level on menopause (average = 67%, sd = 18.41, median = 70, range = 10–100), their knowledge level from 10 years ago (average = 30%, sd = 21.23, median = 27, range = 0–100) and knowledge based on correct responses to objective facts about menopause 56% (sd = 17.69, median = 62.5, range = 12.5–100). The Wilcoxon signed-rank test revealed statistically significant higher levels of rating of current knowledge and knowledge 10 years ago (Z = 10.841, *p* < .001) than knowledge (Z = 9.079, *p* < 01). Knowledge 10 years ago was significantly lower than knowledge (Z = 8.180, *p* < .001). Level of education was positively correlated with current rating of knowledge, knowledge and rating of past knowledge (ρ = 0.19, *p* = .019; ρ = 0.31, *p* < .001 and ρ = 0.20, *p* = .011, respectively).

Participants were split into 3 categories based on self-identified menopausal status: 19 pre-menopausal (average age 45 years), 99 peri-menopausal (average age 51 years) and 44 post-menopausal (average age 56 years). These average ages are comparable with data on typical age of onset and post-menopause.^1^ Descriptive data and statistical tests outcomes can be seen in Table 1. Tests between groups were significant for both current and 10 years knowledge (Z = 18.262, *p* < .001, and Z = 14.072, *p* < .001, respectively) whereas there was no difference between groups for knowledge level. Post-hoc analysis revealed current knowledge was significantly lower in the Pre group to the other groups (Z = 3.977 *p* < .001 and Z = 3.939, *p* < .001) though no significant difference between the Peri and Post groups was observed. For previous knowledge, significant differences were observed between Pre and Post, and Peri and Post (Z = 3.359, *p* < .001 and Z = 2.615, *p* = .009, respectively) though Pre and Peri revealed no significant difference.

**Table 1.** Perceptions of menopause rating knowledge now, knowledge and 10 years ago among pre-, peri-, and post women (average and sd).

	Pre	Peri	Post	KWT	*p* value
Perceived current knowledge level	47.3 (21.7)	69.1* (16.1)	72.2* (16.8)	18.13	<.001
Knowledge level	40.9 (17.8)	51.0 (15.7)	50.7 (13.8)	4.82	.09 ^ns^
Previous knowledge level	20.1 (23.6)	28.1** (18.9)	39.2* (22.7)	14.07	<.001

N.B. Pre = pre-menopausal women, Peri = peri-menopausal women, Post = post-menopausal women, KWT = Kruskal–Wallis H. post-hoc using Mann–Whitney, * = significantly different from pre, ** = significantly different from post.

The number of correct responses to the knowledge questions is presented in Table 2. While some questions had greater awareness, the average age of reaching menopause, bone density loss and the duration of symptoms were poorly understood in the sample.

**Table 2.** The number of correct answers to the knowledge questions.

Knowledge question	*n* correct
Consecutive months without a period to be classified as menopausal	99
Average age women in the United Kingdom reach menopause	17
Blood test used as a menopausal status check	47
Bone density lost in the first 5 years post-menopause	13
Nature of hormone replacement therapy	114
Average duration of menopause symptoms	28
Health risks not associated with menopause	138

N.B. total *n* = 162.

### Sources of information on menopause

Women were asked who they would or had spoken to and where they obtained information regarding menopause. These percentages are presented in Tables 3 and 4. Mothers were the main people who women would speak to for all groups however those going through menopause reported less frequent conversations with this group, instead relying on other female family members. Having gone through menopause, women would be less likely to speak to male family members and not likely to speak to a healthcare professional.

**Table 3.** Who women reported they would discuss menopause with by group.

Person	Pre (*n* = 19) (%)	Peri (*n* = 99) (%)	Post (*n* = 44) (%)
Partner	11	8	14
Friend	11	16	18
Mother	53	30	50
Other female family member	5	22	11
Male family member	16	13	7
Healthcare professional	5	8	0

N.B. Pre = pre-menopausal women, Peri = peri-menopausal women, Post = post-menopausal women.

**Table 4.** Percentage of each source used for information on menopause.

Source of information	Pre (*n* = 19)	Peri (*n* = 99)	Post (*n* = 44)	Total (*n* = 162)
Online searches	42	71	64	65
Healthcare professional	16	59	59	54
Friends	63	83	66	76
Family member	42	32	27	32
Education provider	11	10	5	9
Personal experience	11	61	86	62
Other	32	20	20	22

N.B. Pre = pre-menopausal women, Peri = peri-menopausal women, Post = post-menopausal women.

Sources of information varied across the groups. There was a significant difference in the frequency of conversations with healthcare professionals for those in the Peri and Post compared to Pre (χ^2^_(2)_ = 12.448; *p* = .002), with the Peri group relying on friends significantly more (χ^2^_(2)_ = 6.690; *p* = .035) and understandably, personal experience was a greater source of information for those in the Peri and Post groups than the Pre group (χ^2^_(2)_ = 32.441; *p* < .001).

### Views, beliefs and the hormonal treatment of menopause

Participants were asked to rate how they perceived the time of menopause in a woman’s life. Overall, the rating was slightly negative (Ave = 2.23, median = 2, range 1 = very negative, 5 = very positive). While Pre rated menopause slightly more positively (Ave = 2.32, median = 2) to the other groups (Peri: Ave = 2.19, median = 2 and Post: Ave = 2.27, median = 2), there was no difference between the groups.

**Figure 1.**
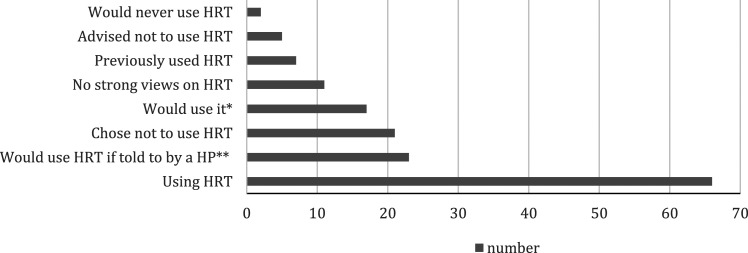
Women’s views on the use of hormone replacement therapy. N.B. H*p* = healthcare professional. * Respondents were mostly pre-menopausal women. ** Respondents were predominantly peri-menopausal women.

The largest proportion of women (41%) were currently using HRT (Figure 1) while women chose either not to use it (13%) or said they would never use it (1%). Between Pre, Peri and Post groups, the statistically significant different responses were ‘I would use it if told to by a healthcare professional’ and ‘currently using’ (*p*=<.05). Significantly differences between Pre and Post also included ‘I chose not to use it’ (*p*=<.05).

### Level of physical activity and awareness of the role of exercise

79% of the sample rated themselves as either somewhat or extremely PA with an average of 3.88 rating (median = 4, range = 1–5). The Pre group rated their level of PA lowest at 3.53 (median = 4) then Peri most active (Ave = 3.96, median = 4) and the Post slightly lower at 3.84 (median = 4) with no statistical differences between groups. Participants view on the role of exercise to impact menopause symptoms was positive (Ave = 4.21, median = 4, range = 1–5). Interestingly, the Peri group felt most positively toward exercise (Ave = 4.33, median = 5) compared to Pre and Post (Ave = 3.89, median = 4 and Ave = 4.07, median = 4, respectively) and the Kruskal–Wallis was significant (H_(2)_ = 6.027, *p* = .049). Peri had a significantly higher rating perceiving there was a role for exercise in managing symptoms compared to the Post group (Z = 2.089, *p* = .037). No other group comparisons were significant.

The most reported barriers were a lack of time (66%), a lack of motivation (51%) and feeling too tired (49%). Lack of social support and lack of knowledge were not typically barriers to exercise (1% each) and few individuals (11%) found no barriers to exercise. Between-group comparison revealed no significant differences for these barriers. Reasons for exercise were principally ‘for general health’ (72%) and ‘to get in shape’ (69%) though few (5%) had received any recommendation to exercise. There were no differences between groups to exercise. Recommendation from healthcare professionals to exercise related to menopause were reported amongst 17% of the sample with 4% unsure. These recommendations were 17% amongst the Peri and 20% for the Post. Only 1 of the 19 Pre menopause women reported having had exercise mentioned by their healthcare professional even though they were within 11 years of passing through menopause.

When asked what aspects of menopause might be impacted by exercise, participants reported that they felt psychological health (98%), muscular strength (93%), bone health (87%) and overall quality of life (85%) would be positively impacted. The three groups were consistent in their views on the positive impact of exercise on menopause. The types of exercise perceived to be beneficial included mixed exercise (55%) or cardiovascular exercise (15%) modes appropriate. However, 19% reported not knowing.

## Discussion

This study assessed the knowledge, sources of knowledge and awareness of menopause amongst women between the ages of 40 and 65 who are pre-, peri- and post-menopausal and gained insight into their beliefs about the role exercise could play during this time.

### Menopause knowledge and awareness

This study found that these women’s estimate of their current knowledge of menopause had improved from 10 years ago. While this change may reflect ageing and/or passing through menopause, the answers on the signs, symptoms and effects did not match those ratings of current knowledge. All indices of knowledge were higher in those post-menopause women, influenced presumably by experience, though there were gaps in knowledge for all groups. However, given the variety of symptoms from pre- to post-menopause and individual experiences may vary to some degree may explain why experience did not strongly link to a higher level of knowledge. Likewise, the various effects of menopause are not always immediately apparent, that is, bone density and fracture risk as loss in calcium reserves can have subtle impact on function which is incremental.^18^ Educational attainment and menopause knowledge were positively but not strongly related. We note that the current sample has relatively highly educated status with only 10% O-level/CSE or lower attainment and that the proportion of individuals with occupations within education and medicine was high.

### Sources of information

One explanation for the level of knowledge discrepancy between self-rating and knowledge maybe the sources of information relied on. Women across all groups relied on ‘friends’ (76%) and online searches (65%) as sources of information, a similar pattern observed by Harper et al.^9^ (friends = 63% and websites 62%). Personal experience (62%) also came ahead of healthcare professionals as a source, and there was a notable discrepancy between the Pre (11%/16%) and the other groups (Peri: 61%/86% and Post: both 59%, respectively). The least used source across all groups was an ‘education provider’ (9%). This implies that women’s knowledge is obtained through informal means generally rather than education or healthcare systems and may explain the level of knowledge observed. This presents a challenge for getting quality information out through formal means especially for those pre-menopausal. Additionally, of those who had interacted with healthcare professionals there was generally frustration at the level of understanding.

### Views of healthcare professional and treatment of menopause

Five themes were identified from participants’ responses: lack of healthcare professional awareness and support; the pros and cons of menopause; the subject more broadly of menopause; perceptions of others and effectiveness of HRT. Typical comments about the theme of healthcare professionals’ awareness included ‘*It’s taken me a few years of tracking symptoms to feel like my doctor is truly listening/believing me’* and a there was ‘*Lack of understanding and support’*. The Fawcett Society survey^19^ of women aged 45–55 who are currently or have previously been peri-menopausal reported 45% had not spoken to their GP surgery about their symptoms a similar number to the current sample. Those who had approached their surgery 31% stated it took several appointments before their GP realised they were experiencing perimenopause.^20^ Even two of the healthcare professions within our sample reported ‘*we are poorly prepared’* and that it is ‘*often undiagnosed’*. These results suggest there is a need to develop healthcare professionals’ awareness and education. Indeed, Armeni’s et al.^19^ survey of medical school curriculum found that 41% of 32 do not incorporate mandatory education concerning menopause.

Hunter and Rendall^21^ observed that menopause is generally a neutral experience in healthy women with earlier studies reporting more negative experiences of the menopause.^22^ However, these early studies typically relied on samples of women seeking health treatment.^21^ Our sample rated menopause a slightly negative time of their life. In addition to the physical symptoms, participants reported menopause is seen as a ‘*taboo subject’*, or a ‘*dirty secret’* and women do not feel it is taken seriously:

“It’s a joke, ‘having a hot flush love’, ‘cotton wool brain again’! Looks of sympathy from some, long looks of confusion from men when glasses steam up during a flush. It’s not taken seriously”.

The lack of knowledge of males was mentioned as a reason for the negative view; ‘*The understanding of husbands can be negative too due to their lack of knowledge’*. Munn et al.^12^ reported that women felt it was essential for men to be educated on menopause as well as a need for better understanding of menopause to reduce ignorance enabling support. Likewise, the Fawcett Society^20^ reported that 41% of women had seen menopause and menopause symptoms *‘treated as a joke by people at work’*; therefore, a universal understanding may assist though since 2019, teaching of the menopause has become compulsory in schools as a part of Relations and Sex Education.

In terms of women’s views on HRT, many women were using it, would use or would use it if advised (41%, 14% and 10%, respectively). These data are broadly in line cumulatively with a web-based survey completed by Cumming et al.^23^ that reported 75% of respondents approved the use of HRT, and 36% felt that the media had exaggerated its potential risks. However, 95% of women would prefer to use alternative therapies prior to HRT.^23^ Results from this study and previous studies have shown that the use of HRT is becoming increasingly more popular and accepted from a period where women were reluctant to use HRT.^24^ HRT was mentioned often by the respondents as a positive aspect of menopause. The common theme amongst women was HRT’s effectiveness in easing symptoms; ‘*Since I have been prescribed HRT, I feel much better, and my health and well-being are much better’*. However, there were also preferences to not be on HRT.

### Knowledge on the role of physical activity

Alternative or adjunct treatments are important for peri- and post-menopausal women who respond poorly to HRT, have side-effects or have a preference towards non-pharmaceutical options.^16^ Our sample had a predominantly positive view on the role of exercise to help manage their symptoms (78%) and reported being PA (79%). A small proportion (17%) reported a healthcare professional had spoken to them about PA and exercise benefits for symptom management and long-term associated risks. From our study, the principal reason in which women exercise is for ‘general health’ (72%) and to ‘stay in shape’ (69%) with the Peri group being most physically active. It is known that obesity and metabolic syndrome are found in women in this period of their life three times more often than before menopause.^25^ The declines of endogenous estrogen, together with physical inactivity, seem to be the major causes of this phenomenon.^26^ Majority of women throughout this report were aware of the positives PA and exercise has on menopause, knowledge on benefits in psychological health (98%), muscular strength (93%), bone health (87%) and overall quality of life (85%). However, many women in the United Kingdom remain physically inactive (exact reasons why require further investigation) common reasons for lack of activity presented in this study were lack of time (66%), lack of motivation (51%) and feeling too tired (49%).

Daley and Thomas^17^ reported that women were more inclined to use non-pharmacological treatments such as exercise for vasomotor menopausal symptoms believing that it reduced the frequency and/or the intensity of their symptoms. Conversely, decreasing their PA levels was linked to their symptoms reappearing.^17^ Dąbrowska-Galas et al.^27^ found moderate and high PA levels in all International Physical Activity Questionnaire domains (work, transport, household and leisure) were associated with lower symptom severity compared to inactive women. Peri-menopausal women reportedly experienced symptoms most severely and therefore benefited most from exercise. Further studies are required to determine adequate exercise dose in reducing symptom severity and to determine methods of reducing physical inactivity in women over this period of life.

### Strengths, limitations and future directions

While these observations of women’s knowledge, attitudes, HRT and the impact of exercise are generally positive, there are caveats. Firstly, the sample was predominantly educated over O-level/CSE. Level of education has been frequently shown to be positively correlated with general health and awareness^28^ and PA levels^29^ Secondly, the proportion of individual’s rating themselves as being PA active is somewhat higher in this sample than would be expected in the general population (55%).^30^ Further directions should attempt to purposefully address these potential biases to obtain a broader perspective.

## Conclusion

This study found that women’s knowledge level is not in line with their perceptions and the explanation may, in part be due to where they are obtaining that information. Knowledge was higher in those peri- and post-menopause but the need to have gone through process without preparation does not benefit the individual approaching menopause. Women were not happy with the level of knowledge healthcare professionals have and it is evident that advancements need to be made in ensuring menopause education is embedded effectively into medical training. Additionally, enhancing social support through broader education in menopause, that is, family members and the recent changes in teaching about of menopause in school may improve menopause awareness. PA as an intervention needs to be investigated further as a prescribed treatment for menopause symptoms and long-term associated risks. Promoting PA to inactive women generally and particularly in this life stage needs to be supported along with studies establishing efficient regimes/doses of PA to assist practitioners inform patients.
